# Evaluation of the Validity of Preemptive Therapy against Cytomegalovirus Disease Based on Antigenemia Assay with a Cutoff of 20 Positive Cells per Two Slides

**DOI:** 10.1371/journal.pone.0073754

**Published:** 2013-09-05

**Authors:** Kana Sakamoto, Hideki Nakasone, Hidenori Wada, Ryoko Yamasaki, Yuko Ishihara, Koji Kawamura, Masahiro Ashizawa, Miki Sato, Kiriko Terasako-Saito, Tomohito Machishima, Shun-Ichi Kimura, Misato Kikuchi, Shinichi Kako, Junya Kanda, Rie Yamazaki, Aki Tanihara, Junji Nishida, Yoshinobu Kanda

**Affiliations:** Division of Hematology, Saitama Medical Center, Jichi Medical University, Saitama, Japan; University Medical Center Utrecht, Netherlands

## Abstract

**Background:**

Preemptive therapy with ganciclovir (GCV) based on the results of a cytomegalovirus (CMV) antigenemia assay is a standard strategy for preventing CMV disease after allogeneic hematopoietic cell transplantation (HCT). However, the appropriate threshold of antigenemia-positive cells for deciding when to start GCV remains unclear.

**Patients:**

This retrospective study included 80 recipients who received HCT from an alternative donor between 2007 and 2011. In 2009, we switched the threshold from 3 (3A group, n=24) to 20 (20A group, n=56) antigenemia-positive cells per two slides for preemptive therapy after HCT from an alternative donor.

**Results:**

Early CMV disease within 100 days after HCT was observed in one patient in the 20A group. Antiviral agents including GCV, val-GCV, and foscarnet were given in 17 (71%) and 36 (64%) patients in the 3A and 20A groups, respectively (p=0.23). In 13 (23%) patients in the 20A group, the initiation of preemptive therapy was avoided because of the change in the cutoff value for CMV antigenemia. However, the total dose of GCV was not different between the two groups. The use of steroid was significantly associated with CMV antigenemia of at least 20 positive cells among patients with low-level antigenemia at the first detection.

**Conclusion:**

The increased threshold up to 20 positive cells for starting preemptive therapy was not associated with a significant increase in CMV disease, but the total dose of GCV was not reduced and there was one early CMV disease in the 20A group. We should explore how to identify patients who are at high risk for increased antigenemia among patients with low-level antigenemia, but at least, preemptive therapy should not be withheld in patients who are already receiving systemic steroid.

## Introduction

Cytomegalovirus (CMV) infection is one of the major complications after allogeneic hematopoietic cell transplantation (HCT). Monitoring of CMV reactivation with a CMV antigenemia [1–4] or a real-time PCR [5,6] assay and preemptive therapy using ganciclovir (GCV) is the standard strategy for preventing CMV disease after allogeneic HCT [7–10]. However, the appropriate threshold number of antigenemia-positive cells for deciding when to start GCV has not been clarified.

Takenaka et al. monitored CMV infection and CMV-associated disease in patients who received related or unrelated donor bone marrow transplantation using CMV antigenemia assay without any prevention or preemptive therapy for CMV [11]. They showed that the incidence of CMV-associated disease was significantly higher in patients with CMV-antigenemia-positive cells in excess of 10 per 50,000 WBCs than in patients with less than 10 positive cells. They utilized HRP-C7 monoclonal antibody instead of C10/C11 that we used, and the 10 positive cells per 50,000 WBCs in their assay correspond to 20 positive cells per two slides in our assay. Therefore, at first, we applied 20 positive cells as the cutoff value for preemptive therapy in HLA-identical related donor transplantation and have been using this cut-off value safely and effectively [1,12]. However, it is not clear whether the same threshold can be applied in HCT from an alternative donor other than a HLA-matched sibling donor.

We previously performed a randomized controlled trial to compare plasma real-time PCR and the antigenemia assay for monitoring CMV reactivation in bone marrow transplantation from HLA-matched unrelated donors [13]. In that study, the threshold was 300 copies /ml in the PCR group and 3 positive cells per 2 slides in the antigenemia group. Early CMV disease was successfully prevented in both groups, but preemptive therapy with GCV was started significantly more often in the antigenemia group (44.2% vs 73.3%, P=0.0089). In addition, the median number of CMV antigenemia-positive cells at the start of GCV was 47 in the PCR group. Therefore, a cutoff value of 3 positive cells per two slides by antigenemia assay might lead to the excessive administration of GCV. Based on these findings, we changed the threshold number of antigenemia-positive cells from 3 to 20 even in HCT from alternative donors. In this study, we compared the clinical course after HCT between two groups with different cut-off values of antigenemia-positive cells.

## Patients and Methods

### Patients

We retrospectively collected data regarding adult patients who received allogeneic HCT from alternative donors at our center from June 2007 to December 2011. A CMV antigenemia-guided preemptive approach for CMV disease had been applied in all of them. Alternative donors included all donors other than HLA-matched related donors. Unrelated PBSCT was not available in Japan during the study period. Exclusion criteria were as follows: patients who received HCT from cord blood or HLA haploidentical donors, patients who received alemtuzumab in the conditioning regimen, patients who failed to achieve neutrophil engraftment, and donor and recipient pairs who were CMV antibody-negative. The mismatched related donors in this study included all the donors who were HLA 1 allele, 1 antigen, or 1 antigen and 1 allele mismatched. These patients were not included in the haploidentical transplantation protocol and treated with a method similar to that for HLA-matched transplantation [14].

### Monitoring with CMV antigenemia and preemptive therapy for CMV infection

A CMV antigenemia assay using MoAb C10/C11 was performed as described previously [15]. Briefly, 1.5 x 10^5^ peripheral blood leukocytes were attached to a slide using a cytocentrifuge and fixed with formaldehyde. The cells were sequentially immunostained with monoclonal antibody C10/11(Clonab CMV; Biotest, Dreieich, Germany), which raised against CMV pp65 antigen, and reacted with goat alkaline phosphatase-labeled anti-mouse immunoglobulin (Mitsubishi Kagaku Iatron Inc, Tokyo, Japan). These cells were analyzed under a light microscope and the results are presented as the sum of the number of positive cells per two slides. An antigenemia assay was performed at least once a week after engraftment. The criterion for engraftment was the achievement of peripheral blood neutrophil count of 0.5 x 10^9^/L or more for 3 consecutive days. Preemptive therapy with GCV or valganciclovir (VGCV) was started when more than the threshold level of CMV-positive cells were detected. In April 2009, we changed the threshold number of antigenemia-positive cells from 3 to 20 per two slides in HCT from alternative donors, whereas it was consistently 20 in HCT from HLA-matched related donors.

As preemptive therapy, the induction dose of GCV or VGCV was 5 mg/kg/day or 900 mg/day, respectively. Preemptive therapy with a low initial dose of GCV has been shown to be effective even in high-risk patients in a previous study [2]. In case of renal dysfunction, the dose was adjusted accordingly [16]. When an increase in the CMV antigenemia level by at least 50% of the previous value was detected, the dose of GCV or VGCV was increased to 10 mg/kg/day or 1800 mg/day, respectively. Conversely, when the number of CMV antigenemia-positive cells decreased to less than 50% of the previous value, the dose of GCV or VGCV was decreased to 5 mg/kg/day or 900 mg/day, respectively. When the number of antigenemia-positive cells declined below the threshold level in patients who were receiving 5 mg/kg/day of GCV or 900 mg/day of VGCV, preemptive therapy was discontinued. When we calculated the cumulative dose of GCV, the original dose before renal adjustment was used for patients with renal dysfunction and VGCV was converted to the corresponding dose of GCV. Previous studies have shown that 900 mg/day of oral VGCV results in an area under the plasma concentration-time curve (AUC) for GCV similar to that of intravenous GCV 5 mg/kg/day [17,18]. In addition, Winston DJ et al. have compared the pharmacokinetics of VGCV and GCV with the same dosage as that in the Pescovitz’s study in post-HSCT patients with stable gastrointestinal GVHD and showed that the mean GCV AUC value was similar [19]. Therefore, we considered that the corresponding dose of 900 mg/day of VGCV was 5 mg/kg/day of GCV. Foscarnet was used in patients with GCV-resistant CMV infection and, in the cumulative-dose calculation, 90 mg/kg/day of foscarnet was considered to be comparable to 10 mg/kg/day of GCV.

### Definitions

We divided alternative donor recipients into 2 groups according to the two different cut-off values of CMV antigenemia-positive cells for preemptive therapy: the 3A-group and the 20A-group. “A” stands for alternative and indicates the donor type. We also used this higher threshold in patients who underwent HCT from HLA-matched related donors (20R-group).

We retrospectively analyzed the incidence of early CMV diseases, defined as CMV diseases occurring before day 100 of HCT. The diagnosis of CMV diseases was made by histopathological examinations. However, CMV retinitis could be diagnosed when characteristic retinal changes were found by ophthalmoscopy. The diagnostic criteria for specific CMV infections have been explained elsewhere [20].

### Statistical analysis

The numbers of days to events were calculated from the date of HCT. Comparisons between groups were performed with Fisher’s exact test for categorical variables and the Mann–Whitney *U*-test and Kruskal-Wallis test for continuous variables. The effect of steroid on the development of CMV antigenemia of at least 20 positive cells per two slide was evaluated with Cox proportional hazards modeling while treating the use of systemic steroid as a time-dependent covariate. Statistical significance was defined as a 2-sided P-value of <0.05. All statistical analyses were performed with EZR (Saitama Medical Center, at http://www.jichi.ac.jp/saitama-sct/SaitamaHP.files/statmedEN.html), which is a graphical user interface for R (The R Foundation for Statistical Computing, version 2.13.0). More precisely, it is a modified version of R commander (version 1.6-3) that was designed to add statistical functions that are frequently used in biostatistics [21].

### Ethics

This study was approved by the institutional review board of Jichi Medical University. Written informed consent was obtained from each patient to be stored in the database and used for research.

## Results

### Patient characteristics

There were 24 and 56 patients in the 3A and 20A groups, respectively. Overall, the median age was 45.5 years. The 20A group included significantly more patients with HLA-mismatched unrelated donors and tended to be older (40 vs. 47.5, P=0.060) and to receive more bone marrow graft (75% vs. 91%, P=0.078, Table 1).

**Table 1:** Patient characteristics.

	**3A**	**20A**	**P value**
**Number**	24	56	
**Median age** (range)	40 (16-59)	47.5 (15-63)	0.060
**Sex**			
Male/Female	12/12	35/21	0.33
**Disease**			0.52
AML	10	27	
ALL	2	8	
MDS/MPD	3	9	
ML/MM	5	9	
sAA	4	3	
**Disease status**			0.86
CR1	6	16	
CR2-4	6	15	
relapse/refractory	5	14	
primary therapy	7	11	
**Performance status**			0.087
0/1	22	56	
2/4	2	0	
**Donor source**			0.078
BM	18	51	
PB	6	5	
**HLA compatibility**			0.0256
Matched unrelated	11	30	
Mismatched unrelated	5	21	
Mismatched related	8	5	
**Conditioning regimen**			
MAC	16	29	0.325
RIC	8	27	
TBI/nonTBI	19/5	40/16	0.59
**Use of ATG**			0.19
Yes	4	3	
No	20	53	
**GVHD prophylaxis**			0.096
CYA-based	23	45	
TAC-based	1	11	
**Recipient / Donor CMV status**			0.24
Pos. / Pos.	14	31	
Pos. / Neg.	5	21	
Neg. / Pos.	4	4	
Missing. / Pos.	1	0	
**Acute GVHD**			0.63
grade 0-1	12	32	
grade 2-4	12	24	
**Use of steroids**			0.45
Yes	17	33	
No	7	23	

AML: acute myeloid leukemia, ALL: acute lymphoblastic lymphoma, MDS: myelodysplastic syndrome, MPD: myeloproliferative disease, ML: malignant lymphoma, MM: multiple myeloma, sAA: severe aplastic anemia, CR: complete response, BM: bone marrow, PB: peripheral blood, MAC: myeloablative conditioning, RIC: reduced-intensity conditioning, ATG: antithymoglobulin, GVHD: graft-versus-host disease, CYA: cyclosporine, TAC: tacrolimus, Pos: positive, Neg: negative

### Incidence of CMV reactivation and preemptive therapy

The incidence of the detection of at least 3 antigenemia-positive cells was not significantly different between the 3A and 20A (71% vs. 84%, p=0.23, Table 2). The incidence of the detection of at least 20 antigenemia-positive cells was significantly higher in the 20A group (63%) compared to the 3A group (29%, p<0.01). In 4 (12%) patients in the 3A group and 12 (21%) in the 20A group, the number of CMV-positive cells exceeded 20 at the first detection.

**Figure 1:**
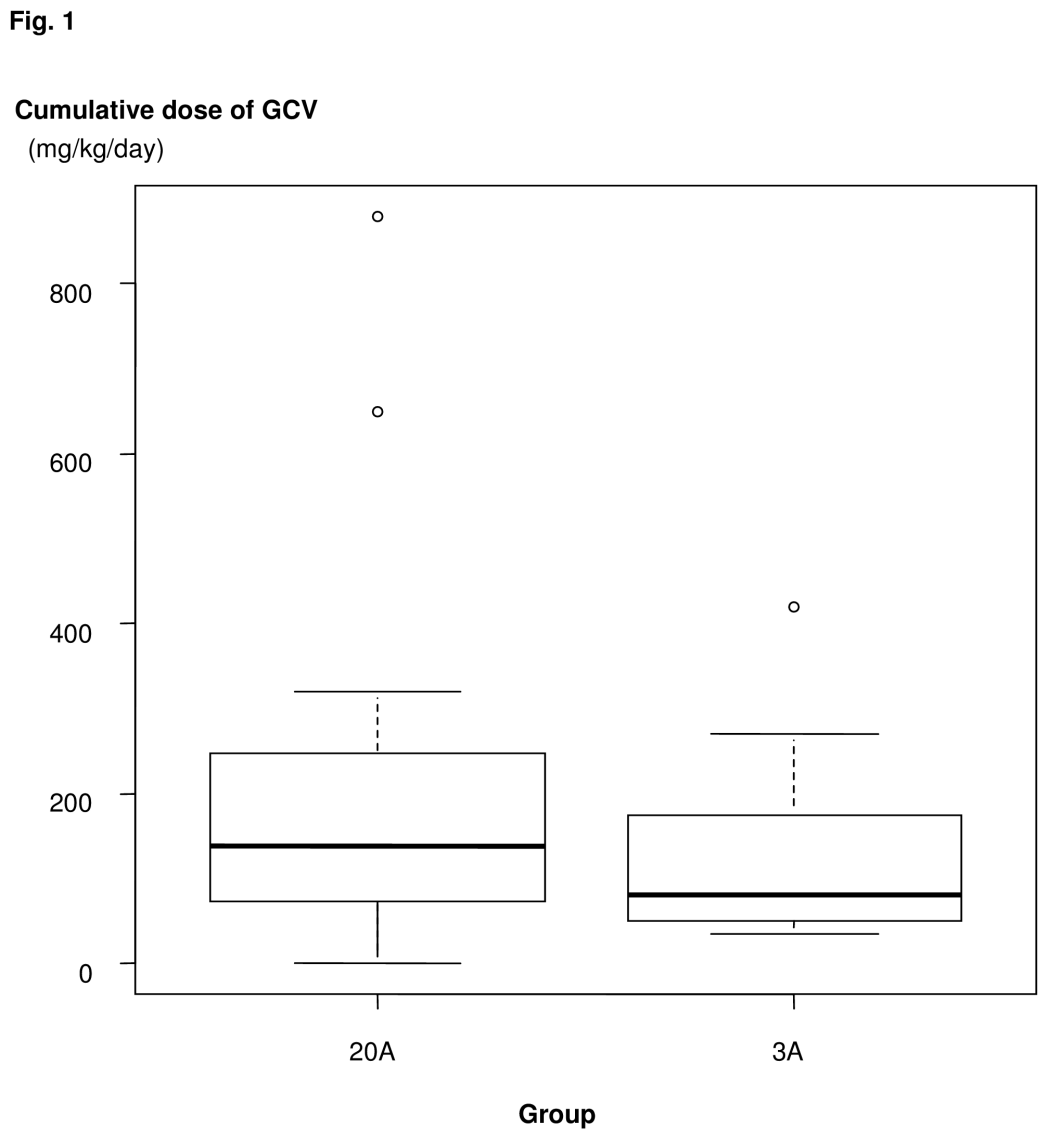
Cumulative dose of ganciclovir (GCV) as preemptive therapy.

Overall, GCV, VGCV, or foscarnet as CMV preemptive therapy or for other reasons was started within 100 days after HCT in 17 (71%) and 36 (64%) patients in the 3A, and 20A groups, respectively (p=0.62, Table 2). The median cumulative doses of GCV within 100 days after HCT were 60 mg/kg/day (range 0 - 420) in the 3A group and 67.5 mg/kg/day (range 0 - 880) in the 20A group (p=0.83, Figure 1). Among the recipients who actually received GCV, the median cumulative doses were 80.0mg/kg/day and 137.5mg/kg/day in the 3A and 20A groups, respectively (p=0.19).

**Table 2:** Positive conversion of CMV antigenemia and preemptive therapy for CMV infection.

	**3A (N=24)**	**20A (N=56)**	**P value**
**Number of patients who developed ≥ 3 positive cells**	17 (71%)	47 (84%)	0.23
**Number of patients who developed ≥ 20 positive cells**	7 (29%)	35 (63%)	0.0077
**Day when number of positive cells became ≥ 3 (median) (range)**	41 (20-71)	41 (16-69)	0.54
**Day when number of positive cells became ≥ 20 (median) (range)**	41 (33-61)	46 (16-75)	0.81
**Number of patients who developed ≥ 20 positive cells at the emergence of positivity**	4 (17%)	12 (21%)	0.77
**GCV/VGCV/FCV started within 100 days from SCT**	17 (71%)	36 (64%)	0.62

Three patients received GCV or foscarnet for possible virus infection, regardless of the CMV antigenemia level. In one case, the therapeutic dose of GCV was started for respiratory failure of unknown etiology because viral pneumonia could not be denied. Eventually, the cause of lung infiltration was considered to be body fluid retention associated with tacrolimus. The number of CMV antigenemia-positive cells was one at that time. In the other two cases, therapeutic doses of GCV or FCV were started for the disturbance of consciousness of unknown etiology because viral encephalopathy could not be denied. In the first case, CMV antigenemia-positive cells were not detected at that time. The etiology of altered mental status could be tacrolimus-induced encephalopathy, but it was not clear even later. In the second case, CMV antigenemia positive cells could not be checked at the onset because the neutrophil count was too low to perform the assay. The cause of disturbance of consciousness was considered to be bacterial meningitis and posterior reversible encephalopathy syndrome. If we excluded these 3 patients, GCV or VGCV was given in 17 (71%) and 34 (61%) patients in the 3A and 20A groups, respectively (p=0.45). The cumulative dose of GCV was also not significantly different between these groups (data not shown).

Preemptive therapy was withheld in 35 patients in the 20A group at the first detection of CMV antigenemia of between 3 and 19 positive cells per two slides. In 13 of these 35 patients, preemptive therapy was avoided because the number of positive cells did not reach 20 (AVOID group). The other 22 patients received preemptive therapy a median of 7 days later, since the number of antigenemia-positive cells eventually reached at least 20 (DELAY group). The cumulative dose of GCV in the DELAY group was 135 mg/kg/day (median, 45-880), which was higher than that in patients in the 3A group who received GCV at the first detection of CMV antigenemia of between 3 and 19 positive cells per two slides (80 mg/kg/day, P=0.15), although this difference was not statistically significant.

There were no significant differences in the patient characteristics between the AVOID group and the DELAY group, including sex, background disease, CMV status, conditioning regimen, use of TBI or ATG, or the presence of grade 2 to 4 acute GVHD, except that the DELAY group included significantly more patients who used systemic steroids within 100 days after HCT (31% vs. 68%, p=0.043, Table 3). To evaluate the effect of steroid more accurately, we performed Cox proportional hazards modeling while treating the use of steroid as a time-dependent covariate. The use of steroid was associated with a significantly higher incidence of CMV antigenemia of at least 20 positive cells per two slides, with a hazard ratio of 4.63 (95% confidence interval 1.89-11.4, P=0.00081).

**Table 3:** Patient characteristics in the AVOID group and the DELAY group.

	**AVOID (N=13)**	**DELAY (N=22)**	**P value**
**Median age** (range)	57	42.5	0.47
**Sex**			
Male/Female	9/4	12/10	0.49
**Disease**			
AML	6	12	0.39
ALL	4	2	
MDS/MPD	2	2	
ML/MM	1	3	
sAA	0	3	
**Disease status**			
CR1	3	7	0.48
CR2-4	3	8	
relapse/refractory	4	2	
primary therapy	3	5	
**Performance status**			
0/1	13	22	-
2/4	0	0	
**Conditioning regimen**			
MAC	5	14	0.18
RIC	8	8	
**TBI regimen**			
Yes	9	16	1.00
No	4	6	
**Use of ATG**			
Yes	0	3	0.28
No	13	19	
**Recipient / Donor CMV status**			
Pos. / Pos.	8	13	1.00
Pos. / Neg.	5	8	
Neg. / Pos.	0	1	
**Acute GVHD**			
Grade 0-1	8	10	0.49
Grade 2-4	5	12	
**Use of steroids within 100 days after HSCT**			
Yes	4	15	0.043
No	9	7	

AML: acute myeloid leukemia, ALL: acute lymphoblastic lymphoma, MDS: myelodysplastic syndrome, MPD: myeloproliferative disease, ML: malignant lymphoma, MM: multiple myeloma, sAA: severe aplastic anemia, CR: complete response, MAC: myeloablative conditioning, RIC: reduced intensity conditioning, ATG: antithymoglobulin, GVHD: graft versus host disease, Pos: positive, Neg: negative

### Early CMV diseases

One patient in the 20A group was diagnosed with CMV disease, and there was no CMV disease in the 3A group. The patient in the 20A group was a 57-year-old male with relapsed follicular lymphoma who received a fludarabine-based conditioning regimen followed by HLA-mismatched related peripheral blood stem cell transplantation. The patient developed steroid-refractory acute GVHD and required prolonged steroid administration. A ground glass appearance with unknown etiology was found in both lungs in the chest CT on day 19, which was 1 week after engraftment. The first antigenemia-positive cells were recorded on day 26 (15 per two slides) and the number increased to 96 on day 35. Preemptive therapy was started immediately, but the number of CMV-positive cells jumped to 456 on day 42 and the dose of GCV was increased to 10 mg/kg. The patient became febrile from day 42 and the oxygenation level became worse. The patient died on day 45. The autopsy showed inclusion bodies of CMV in the lungs and the intestine.

With regard to late CMV disease, one case of CMV enteritis (day 164) and one case of retinitis (day182) were observed in the 20A group. Both were treated with GCV and improved.

### Patients who received HCT from HLA-matched related donors

We also collected data of 39 patients who received HCT from HLA-matched related donors (20R-group) for comparison with the 20A-group. The incidence of CMV reactivation (46%), GCV/VGCV/foscarnet use (36%), and the median cumulative dose of GCV (0.0 mg/kg/day, range 0.0-350) in the 20R group were significantly lower than those in the 3A and 20A groups (data not shown). Early CMV disease was not found in the 20R group.

## Discussion

We retrospectively analyzed the safety of increasing the cutoff value of CMV antigenemia-positive cells from 3 to 20 in preemptive therapy for CMV disease. As expected, 23% of the patients with a high cutoff value could avoid GCV administration without a significant increase in early CMV disease. However, the cumulative dose of GCV in the 20A group was not significantly reduced compared to that in the 3A group. This could be explained by the relatively high cumulative dose of GCV used in the DELAY group.

It is difficult to decide upon an appropriate cutoff value for preemptive therapy. The current study showed that the unnecessary administration of preemptive therapy could be avoided by increasing the cutoff value in a subset of patients with positive antigenemia, but other patients eventually required preemptive therapy with at least a one-week delay. Although there was no statistically significant evidence that delaying in starting preemptive therapy induced an increase in incidence of CMV disease, delaying treatment provided no benefit to patients, since the cumulative dose of GCV was not decreased. Therefore, it is important to be able to identify patients who are at high risk for a subsequent increase in antigenemia-positive cells. There was no difference in the background characteristics between the AVOID group and the DELAY group. However, the use of systemic steroid was associated with a significantly higher incidence of CMV antigenemia of at least 20 positive cells per two slides, with a hazard ratio of 4.63. Therefore, preemptive therapy should be started as soon as systemic steroid is started, whereas patients who do not require systemic steroids could be good candidates for withholding preemptive therapy in the presence of low-level CMV antigenemia. In fact, more than half (56%) of the patients who did not receive steroid could avoid preemptive therapy with an increase in the threshold of antigenemia-positive cells.

Although there was no significant difference in the incidence of CMV disease, one patient in the 20A group developed early CMV disease. He developed interstitial pneumonia before the first detection of positive antigenemia, and CMV disease was diagnosed by autopsy. Therefore, we could not determine the onset of CMV pneumonia. This patient developed severe acute GVHD and was heavily treated with high-dose corticosteroids, and therefore, the development of CMV pneumonia might be a later event. However, in such patients, antiviral agents should be started earlier.

In conclusion, the increased threshold up to 20 positive cells for starting preemptive therapy was not associated with a significant increase in CMV disease. However, there was one case of early fatal CMV disease that was possibly related to the increase in the cut-off value. In addition, delaying the start of preemptive therapy did not result in the decreased use of GCV. On the other hand, subsets of patients could avoid the unnecessary use of GCV. We should explore how to identify patients who are at high risk for increased antigenemia at the first detection of low levels of positive cells. At least, preemptive therapy should not be withheld in patients who are already receiving systemic steroid.
